# The ethanol extract of *Aquilariae Lignum* ameliorates hippocampal oxidative stress in a repeated restraint stress mouse model

**DOI:** 10.1186/s12906-017-1902-1

**Published:** 2017-08-10

**Authors:** Hyun-Yong Lee, Jin-Seok Lee, Hyeong-Geug Kim, Won-Yong Kim, Seung-Bae Lee, Yung-Hyun Choi, Chang-Gue Son

**Affiliations:** 1 College of Korean Medicine, Daejeon University, 62, Daehak-ro, Dong-gu, Daejeon, 34520 Republic of Korea; 2 Liver and Immunology Research Center, Institute of Traditional Medicine and Bioscience of Daejeon University, 176-9, Daeheung-ro, Jung-gu, Daejeon, 34929 Republic of Korea; 3 Department of Biochemistry, College of Korean Medicine, Dong-Eui University, 52-57, Yangjeong-ro, Busanjin-gu, Busan, 47227 Republic of Korea

**Keywords:** Repeated restraint stress, Brain oxidative stress, Hippocampus, Microglia, *Aquilariae lignum*

## Abstract

**Background:**

Chronic stress contributes to the development of brain disorders, such as neurodegenerative and psychiatric diseases. Oxidative damage is well known as a causative factor for pathogenic process in brain tissues. The aim of this study is to evaluate the neuroprotective effect of a 30% ethanol extract of *Aquilariae Lignum* (ALE) in repeated stress-induced hippocampal oxidative injury.

**Methods:**

Fifty BALB/c male mice (12 weeks old) were randomly divided into five groups (*n* = 10). For 11 consecutive days, each group was orally administered with distilled water, ALE (20 or 80 mg/kg) or *N*-acetylcysteine (NAC; 100 mg/kg), and then all mice (except unstressed group) were subjected to restraint stress for 6 h. On the final day, brain tissues and sera were isolated, and stress hormones and hippocampal oxidative alterations were examined. We also treated lipopolysaccharide (LPS, 1 μg/mL)-stimulated BV2 microglial cells with ALE (1 and 5 μg/mL) or NAC (10 μM) to investigate the pharmacological mechanism.

**Results:**

Restraint stress considerably increased the serum levels of corticosterone and adrenaline and the hippocampal levels of reactive oxygen species (ROS), nitric oxide (NO), and malondialdehyde (MDA). ALE administration significantly attenuated the above abnormalities. ALE also significantly normalized the stress-induced activation of astrocytes and microglial cells in the hippocampus as well as the elevation of pro-inflammatory cytokines, such as tumor necrosis factor-alpha (TNF-α) and interleukin-1 beta (IL-1β). The in vitro assay outcome supplemented ALE could dramatically block NF-κB activation in microglia. The anti-oxidative stress effects of ALE were supported by the results of antioxidant components, 4-hydroxynonenal (4-HNE), NADPH oxidase 2 (NOX2), inducible nitric oxide synthase (iNOS) and NFE2L2 (Nrf2) in the hippocampal tissues.

**Conclusions:**

We firstly demonstrated the neuroprotective potentials of *A. Lignum* against hippocampal oxidative injury in repeated restraint stress. The corresponding mechanisms might involve modulations in the release of ROS, pro-inflammatory cytokines and stress hormones.

## Background

Stress is generally defined as a homeostatic disruption from actual or implied threats [1]. After the recognition of stressors, the brain mediates integrative stress-coping responses via the hypothalamic-pituitary-adrenal (HPA) axis, which accompanies the release of stress hormones, including glucocorticoids and catecholamines [2]. Under chronic stress, these stress hormones are significantly elevated, affecting the development or progress of mental and physical disorders, such as neurodegenerative, cardiovascular, and cancerous diseases [3–5].

Brain tissue is a main target of stress, which is somewhat related to oxidative injury [6, 7]. A few studies have revealed that chronic stress can increase the production of free radicals in the brain via the activation of NADPH oxidase (NOX) [8, 9]. The brain is prone to oxidative stress due to its large consumption of oxygen [10], low levels of antioxidants [11], and high contents of iron and polyunsaturated fatty acids [12]. In particular, the hippocampus contains high levels of glucocorticoid receptors and thus, is vulnerable to excessive glucocorticoid exposure or chronic stress [13, 14]. Stress-induced hippocampal oxidative injury is known to be closely related with cognitive impairment and emotional distress [15, 16].

Herbs have gained increased prominence as pharmaceutical agents for preventing brain oxidative stress or neurodegenerative disorders [17–19]. *Aquilariae Lignum* (沉香), also called agarwood, and its rich aromatic compounds have been used in Southeast and East Asian regions for soothing anxiety, pain or inflammation [20, 21]. Recent studies have shown anti-oxidative, anti-inflammatory, and neuroleptic capacities of *A. Lignum* [22–24]. Furthermore, its molecular derivative is known to relieve neuropsychiatric symptoms via modulating central monoamine neurotransmitters in several brain regions [25]. In our preliminary in vitro study, *A. Lignum* extracts significantly protected HT22 hippocampal neuronal cell line from glutamate excitotoxicity, which implies its neuropharmacologial action in stress-related brain disorders.

Therefore, we hypothesized that *A. Lignum* could be a potential candidate against stress-related brain oxidative injury. The current study used a repeated restraint stress mice model to investigate the effects of *A. Lignum* on oxidative distortions, particularly in the hippocampal region, and a supplementary in vitro assay using BV2 murine microglial cell line to explore the pharmacological mechanism.

## Methods

### Preparation of Aquilariae lignum

*Aquilariae Lignum* of the *Aquilariae malaccensis* species was obtained from an herbal pharmaceutical company (Dae Han Bio Pharm Inc., Gyeonggi-do, Korea). An ethanol extract of *A. Lignum* (ALE) was prepared as follows: 10 g of dried *A. Lignum* was pulverized with a grinder and mixed with 100 mL of 30% ethanol on a moving shaker (150 rpm) for 72 h at room temperature (RT). After centrifuging the suspension, the supernatant was collected and filtered through a 300-mesh, 50-nm filter paper (Advantec, Tokyo, Japan). The filtrate was concentrated in a rotary evaporator and lyophilized. The final extraction yield was 6.42% (*w*/w). The extract was dissolved in distilled water before use and the remainder was stored at −80 °C for future use.

### Fingerprinting analysis of ALE

We conducted ultra-high-performance liquid chromatography–tandem mass spectrometry (UHPLC–MS/MS) on ALE to determine its reproducibility and chemical composition. Briefly, a 5-mg aliquot of ALE was dissolved in 1 mL of 90% methanol, and the solution was filtered (0.45-μm). Then, the ALE sample was subjected to UHPLC–MS using an LTQ Orbitrap XL linear ion-trap MS system (Thermo Scientific Co., San Jose, CA, USA) equipped with an electrospray ionization source. Separation was performed on an Accela UHPLC system using an Acquity BEH C18 column (1.7 μm, 2.1 × 150 mm; Waters, Milford, MA, USA). The mobile phase conditions were prepared as follows: (A) was distilled water and (B) was acetonitrile, both of which contained 0.1% formic acid. The column was eluted at a flow rate of 0.4 mL/min with the following gradients: 0 to 1 min, 5% B (isocratic); 1 to 20 min, 5 to 70% B (linear gradient); 20 to 24 min, 70 to 100% B (linear gradient); and 24 to 27 min, 100% B (isocratic). ALE was detected using a photodiode array at 200–700 nm. The full-scan mass spectra were acquired at 150–1500 *m/z* in positive ion mode. The Orbitrap analyzer was used for high-resolution mass data acquisition with a mass resolving power of 30,000 FWHM at 400 *m/z*. Tandem mass (MS/MS) spectra were acquired in data-dependent mode by collision-induced dissociation.

### Reagents and chemicals

The following reagents were obtained from Sigma (St. Louis, MO, USA): 4-amino-3-hydrazino-5-mercapto-1,2,4-triazole (Purpald), 1-chloro-2,4-dinitrobenzene (CDNB), N,N-diethyl-p-phenylenediamine (DEPPD), 5,5-dithio-bis-2-nitrobenzoic acid (DTNB), ferrous sulfate, potassium phosphate, reduced glutathione (GSH), glutathione reductase (GSH-Rd), L-glutathione oxidized disodium salt (GSSG), β-nicotinamide adenine dinucleotide phosphate-reduced form (β-NADPH), and 1,1,3,3-tetraethoxypropane (TEP). Other reagents were obtained from the following manufacturers: anti-4-hydroxynonenal (4-HNE), NADPH oxidase 2 (NOX2), inducible nitric oxide synthase (iNOS), NF-E2-related factor 2 (Nrf2), NF-kappa B p65 (NF-κB p65), β-actin and Lamin B1 antibodies, and horseradish peroxidase (HRP)-conjugated horseradish peroxidase secondary antibody for western blotting (Abcam, Cambridge, MA; Thermo Scientific; and Santa Cruz Biotechnology, Santa Cruz, CA, USA), NE-PER® Nuclear and Cytoplasmic Extraction Reagents for separating cytoplasmic and nuclear proteins (Thermo scientific), anti-glial fibrillary acidic protein (GFAP) and ionized calcium-binding adapter molecule 1 (Iba1) antibodies, and a biotinylated goat anti-rabbit secondary antibody for immunohistochemical staining (Dako, Hamburg, Germany; Wako, Osaka, Japan; Vector Lab., Burlingame, CA, USA), Dulbecco’s modified Eagle’s medium (DMEM), fetal bovine serum (FBS), penicillin, streptomycin and trypsin-ethylenediaminetetra acetic acid (EDTA) solution for cell cultures and in-vitro experiments (Welgene, Gyeongsangbuk-do, Korea), Triton X-100, 4′,6-diamidino-2-phenylindole (DAPI) and a goat anti-rabbit Alexa Fluor® 488-conjugated secondary antibody for cellular immunofluorescent staining (Amresco LLC, Solon, OH, USA; Sigma; Abcam), Bovine serum albumin (GenDEPOT, Barker, TX, U.S.A), thiobarbituric acid (TBA; Lancaster Co., Lancashire, England), H_2_O_2_, (Junsei Chemical Co., Ltd., Tokyo, Japan), *n*-butanol (J.T. Baker, Mexico City, Mexico), a 1 M Tris-HCl solution (pH 7.4) and a 500 mM ethylene diaminetetraacetic acid (EDTA) solution (pH 8.0; Bioneer, Daejeon, Republic of Korea).

### Animals and experimental design

Fifty specific pathogen-free BALB/c male mice (12 weeks old; 26–28 g) were purchased from Dae Han Bio Pharm Inc. They were housed in a room maintained at 23 ± 2 °C with a 12 h light-dark cycle and fed with food (Cargill Agri Purina, Gyeonggido, Korea) and water ad libitum. After acclimation for 1 week, the mice were randomly divided into five groups (*n* = 10): unstressed, control, ALE (20 or 80 mg/kg), and *N*-acetylcysteine (NAC) groups (100 mg/kg, as a positive control). Each group was orally administered with distilled water (unstressed and control group), ALE or NAC every 11 days. One hour after the oral administration, all mice except those in the unstressed group were subjected to restraint stress through confinement inside 50-mL conical tubes (with 0.5 cm air holes for breathing) for 6 h without access to food or water, according to a previous study [26]. The restraint stress model was conducted from 11:00 to 17:00 daily.

The ALE dosage was based on prescreening results of in vitro assays and clinical experiences. The protocol was approved by the Institutional Animal Care and Use Committee (IACUC) of Daejeon University (DJUARB2016–029) and was conducted in accordance with the Guide for the Care and Use of Laboratory Animals published by the United States National Institutes of Health (NIH).

### Preparation of brain tissues and sera

Upon the end of the final restraint stress, the mice were immediately sacrificed under ether anesthesia. Blood and brains were collected following IACUC criteria. Then, the blood was centrifuged at 3000×g for 15 min at 4 °C to separate the sera. Three brains from each group were fixed in 4% paraformaldehyde solution for immunohistochemical staining. The hippocampal regions were isolated from the remaining seven brains of each group. Then, the hippocampal regions were homogenized in radioimmunoprecipitation assay (RIPA) buffer for biochemical analysis and western blotting or in 1.15% KCl for malondialdehyde (MDA) formation assays. The hippocampus homogenates and sera were stored at −80 °C. Protein concentrations of the hippocampus were determined using a Bicinchoninic Acid Protein Assay kit (Sigma).

### Determination of corticosterone and adrenaline

Serum corticosterone levels were measured using commercially available enzyme immunoassay (EIA) kits (Corticosterone EIA Kit, Arbor Assays, Ann Arbor, MI, USA). Serum adrenaline levels were determined using a commercial kit (Adrenaline Research ELISA, LDN, Nordhorn, Germany). Absorbances were measured at 450 and 570 nm using a UV spectrophotometer (Molecular Devices, Sunnyvale, CA, USA).

### Determination of reactive oxygen species (ROS) and nitric oxide (NO) levels

Total ROS levels in the hippocampus and serum were determined as previously described [27]. Briefly, the hippocampal homogenates and sera were prepared with sodium acetate buffer (0.1 M, pH 4.8). After incubation at 37 °C for 5 min, a DEPPD (10 mM)/ferrous sulfate solution (4.37 μM) mixture (1:25) was added. Absorbance was measured at 505 nm using a UV spectrophotometer, and the results were calculated using an H_2_O_2_ standard curve.

The hippocampal NO level was measured using the Griess reagent method [28]. The hippocampal homogenates were mixed with Griess reagent (1% sulfanilamide, 0.1% N-(1-naphthyl) ethylenediamine hydrochloride, and 2.5% phosphoric acid). After incubating at 37 °C for 20 min, the product absorbance was measured at 540 nm using a UV spectrophotometer.

### Determination of malondialdehyde (MDA) level

The hippocampal MDA level was determined using the thiobarbituric acid reactive substance (TBARS) method [29]. The hippocampal tissues were homogenized on ice-cold 1.15% KCl buffer. The homogenates were mixed with 1% phosphoric acid and 0.67% of thiobarbituric acid (TBA). After incubating at 100 °C for 45 min, the mixtures were placed on ice for 5 min and *n*-butanol was added. The homogenates were centrifuged at 3000×g for 15 min at 4 °C, and absorbances at 535 and 520 nm were measured using a UV spectrophotometer. The concentration of TBARS was calculated using a TEP standard curve.

### Immunohistochemical staining analysis

Immunohistochemical analyses were performed to assess the activations of microglial cells and astrocytes with ionized calcium-binding adapter molecule 1 (Iba1) and glial fibrillary acidic protein (GFAP) in the hippocampus. After immersing in a fixative solution for 4 h, the brain tissues were cryoprotected for 24 h in 10, 20, and 30% sucrose, alternately. Then, the tissues were embedded in tissue-freezing medium with liquid nitrogen and cut into coronal frozen sections (35-μm) using a Leica CM3050 cryostat (Leica Biosystems, Buffalo Grove, IL, USA). The sections were stored in the anti-freeze buffer.

Free-floating sections were subjected to endogenous peroxidase quenching with 1% H_2_O_2_ in phosphate-buffered saline (PBS), followed by treatment with blocking buffer (5% normal chicken serum and 0.3% Triton X-100 in PBS) for 1 h. Then, the sections were incubated with Iba1 (1:200, 019–19,741, Wako) or GFAP (1:200, Z0334, Dako) antibodies overnight at 4 °C. After washing four times with PBS, the sections were subjected to a biotinylated goat anti-rabbit (1:400, BA-1000, Vector Lab.) secondary antibody for 2 h and exposed to an avidin-biotin peroxidase complex (ABC; PK-4001, VECTASTAIN ABC HRP Kit, Vector Lab.) for 1 h. The peroxidase activity was visualized using stable 3,3′-diaminobenzidine (DAB; D5637-1G, Sigma) and 3-amino-9-ethylcarbazole (AEC; SK-4200, Vector Lab.). All immunoreactions were observed using an Axio-phot microscope (Carl Zeiss, Oberkochen, Germany) and the results were quantified using ImageJ version 1.46 (NIH, Bethesda, MD, USA).

### Determination of tumor necrosis factor (TNF)-α and interleukin (IL)-1β levels

Hippocampal TNF-α levels were determined using a commercially available EIA kit (Mouse TNF ELISA Set II, BD Biosciences, San Diego, CA, USA). Hippocampal IL-1β levels were also measured using a commercial kit (Mouse IL-1β/IL-1F2 Duoset ELISA, R&D Systems, Minneapolis, MN, USA). Absorbances at 450 and 570 nm were measured using a UV spectrophotometer.

### Western blot analysis

Hippocampal expressions of 4-hydroxynonenal (4-HNE)-modified protein, NADPH oxidase 2 (NOX2), inducible nitric oxide synthase (iNOS), NFE2L2 (Nrf2) and β-actin proteins were analyzed by western blot. The proteins from homogenates were separated by 10% polyacrylamide gel electrophoresis and transferred to polyvinylidene fluoride (PVDF) membranes. After blocking in 5% skim milk for 1 h, the membranes were probed overnight at 4 °C with primary antibodies (4-HNE, NOX2, iNOS, Nrf2 and β-actin). Then, the samples were washed three times and incubated for 2 h with HRP-conjugated anti-rabbit or anti-mouse antibody. Western blots were visualized using an enhanced chemiluminescence (ECL) advanced kit. The protein expression was semi-quantified using ImageJ (NIH).

### Determination of superoxide dismutase (SOD) and catalase

Hippocampal SOD activity was determined using an SOD assay kit (Dojindo Laboratories; Kumamoto, Japan). Absorbance was measured at 450 nm using a UV spectrophotometer, and the results were calculated using dilutions of bovine erythrocyte SOD (Sigma) ranging from 0.01–50 units/mL.

Hippocampal catalase activity was analyzed as previously described [30]. The hippocampal homogenates were mixed with phosphate buffer (250 mM, pH 7.0), methanol (12 mM), and H_2_O_2_ (44 mM) in sequence. After incubation at RT for 20 min, the reaction was stopped by the addition of Purpald solution (22.8 mM Purpald in 2 N potassium hydroxide). The mixture was incubated at 25 °C for 20 min; then, potassium periodate (65.2 mM in 0.5 N potassium hydrate) was added. Absorbance at 550 nm was measured using a UV spectrophotometer, and the results were calculated using a catalase standard curve.

### Determination of total glutathione (GSH) content and glutathione S-transferase (GST) activity

Total GSH contents in the hippocampus were determined as previously described [31]. Hippocampal homogenates were combined with an NADPH (0.3 mM)/DTNB (4 mM) mixture (7:1). Then, a glutathione reductase (GSH-Rd) solution (0.06 units) was added. Absorbance at 405 nm was measured using a UV spectrophotometer, and the GSH contents were calculated using a reduced glutathione standard curve.

The hippocampal GST activity was determined using a commercial assay kit (GST Assay Kit, Sigma). Absorbance at 340 nm was measured using a UV spectrophotometer (Molecular Devices). Enzyme activity was calculated using the following formula: Enzyme activity (unit/mL) = [(∆A_340_)/min × 0.2 × dilution factor]/(5.3 mM^−1^ cm^−1^ × volume of enzyme sample tested).

### Immunofluorescent staining and western blot analysis in BV2 cell line

BV2 murine microglial cells were cultured DMEM supplemented with 10% FBS and 1% penicillin-streptomycin at 37 °C in a humidified incubator with 5% CO_2_. In all experiments, BV2 cells were pre-treated with ALE (1 and 5 μg/mL) or NAC (10 μM) for 3 h before exposure to Gram-negative lipopolysaccharide bacteria (LPS, 1 μg/mL).

For immunofluorescent staining, BV2 cells were plated in 24-well plates at a density of 2 × 10^5^ cells/well for 24 h and washed. Then, the cells were fixed with 4% paraformaldehyde for 10 min, permeabilized with 0.1% Triton X-100 for 10 min, and blocked with 1% bovine serum albumin (BSA) in PBS with Tween 20 (PBST) for 30 min. The cells were incubated in diluted NF-κB p65 antibody in PBST (1:100) for 3 h at RT. After washing, they were incubated with Alexa Fluor® 488-conjugated secondary antibody for 1 h in the dark. The nuclei were counterstained with DAPI solution. Fluorescent images were observed under an Axio-phot microscope (Carl Zeiss).

The cytosolic and nucleic proteins of BV2 cells were separated using NE-PER® Nuclear and Cytoplasmic Extraction Reagents (Thermo scientific) according to the manufacturer’s instruction. Then, western blot analysis was conducted to explore nuclear and cytoplasmic expression of NF-κB p65, and the results were semi-quantified using ImageJ (NIH).

### Statistical analysis

All data are expressed as the mean ± standard deviation (SD). Significant differences between groups were evaluated by one-way analysis of variance (ANOVA) followed by post hoc multiple comparisons with Fisher’s LSD *t*-test using IBM SPSS statistics software, ver. 20.0 (SPSS Inc., Chicago, IL, USA). Differences at *P* < 0.05 or *P* < 0.01 were considered significant.

## Results

### Compositional analysis of ALE

**Fig. 1:**
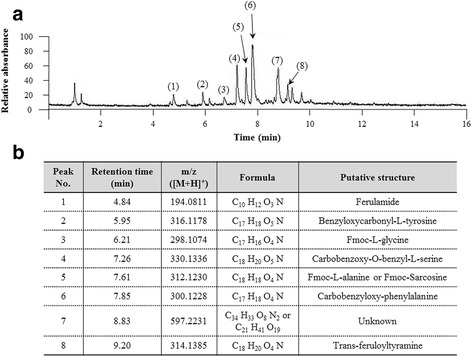
Fingerprinting analysis of ALE. ALE was subjected to UHPLC-MS/MS and a chromatogram was obtained at a wavelength of 254 nm (**a**). The putative chemical structure of each compound was determined using HPLC-MS database (**b**)

In the fingerprinting analysis of ALE, a total eight of major peaks appeared at 4.84, 5.95, 6.21, 7.26, 7.61, 7.85, 8.83, and 9.20 min of retention time under a UV wavelength of 254 nm (Fig. 1a). The chemical compounds of each peak were identified using high-resolution mass spectrometry. The identified chemical formulae were assumed to be C_10_H_12_O_3_N (ferulamide), C_17_H_18_O_5_N (benzyloxycarbonyl-L-tyrosine), C_17_H_16_O_4_N (Fmoc-L-glycine), C_18_H_20_O_5_N (carbobenzoxy-O-benzyl-L-serine), C_18_H_18_O_4_N (Fmoc-L-alanine or Fmoc-Sarcosine), C_17_H_18_O_4_N (carbobenzyloxy-phenylalanine), C_34_H_33_O_8_N_2_ or C_21_H_41_O_19_ (unknown), and C_18_H_20_O_4_N (trans-feruloyltyramine), respectively (Fig. 1b).

### Effects on stress hormones

**Fig. 2:**
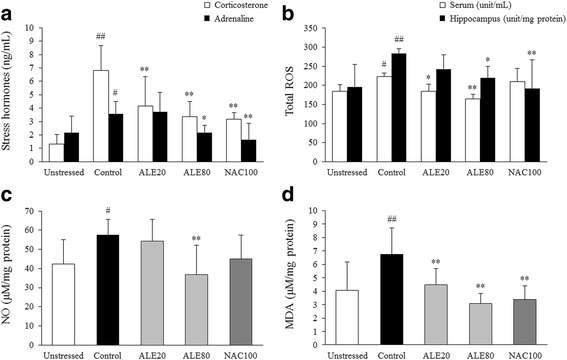
Stress hormones and oxidative stress biomarkers. Corticosterone and adrenaline levels in the serum (**a**), ROS levels in both serum and hippocampus (**b**), and NO (**c**) and MDA (**d**) levels in the hippocampus were determined using ELISA. The data are expressed as the means ± SD (*n* = 7 for hippocampi and = 10 for sera). ^#^ *P* < 0.05 and ^##^ *P* < 0.01 compared with the unstressed group; ^*^ *P* < 0.05 and ^**^ *P* < 0.01 compared with the control group

Repeated restraint stress remarkably increased serum corticosterone and adrenaline levels by 5.2- and 1.6-fold compared with the unstressed group, respectively. ALE treatment significantly reduced the secretion of corticosterone (*P* < 0.01) and adrenaline (*P* < 0.05 for only 80 mg/kg group), respectively (Fig. 2a). NAC treatment similarly decreased the serum levels of these stress hormones.

### Effects on ROS, NO and MDA levels in hippocampus

In the hippocampus region, repeated restraint stress induced the overproduction of free radicals by 1.4-fold for both total ROS and NO compared with the unstressed group, which was significantly ameliorated by ALE (80 mg/kg) treatment (*P* < 0.05 for ROS and *P* < 0.01 for NO, Fig. 2b and c). Lipid peroxidation, measured with MDA level, was notably increased by 1.6-fold in the hippocampus after restraint stress, whereas it was significantly attenuated by ALE treatment when compared with the control group (*P* < 0.01, Fig. 2d). The stress-induced elevation of serum ROS level was also significantly reduced by ALE treatment (*P* < 0.05 or <0.01, Fig. 2b), and NAC treatment showed similar effects on hippocampal ROS and MDA levels.

### Immunohistochemical analysis in the hippocampus

**Fig. 3:**
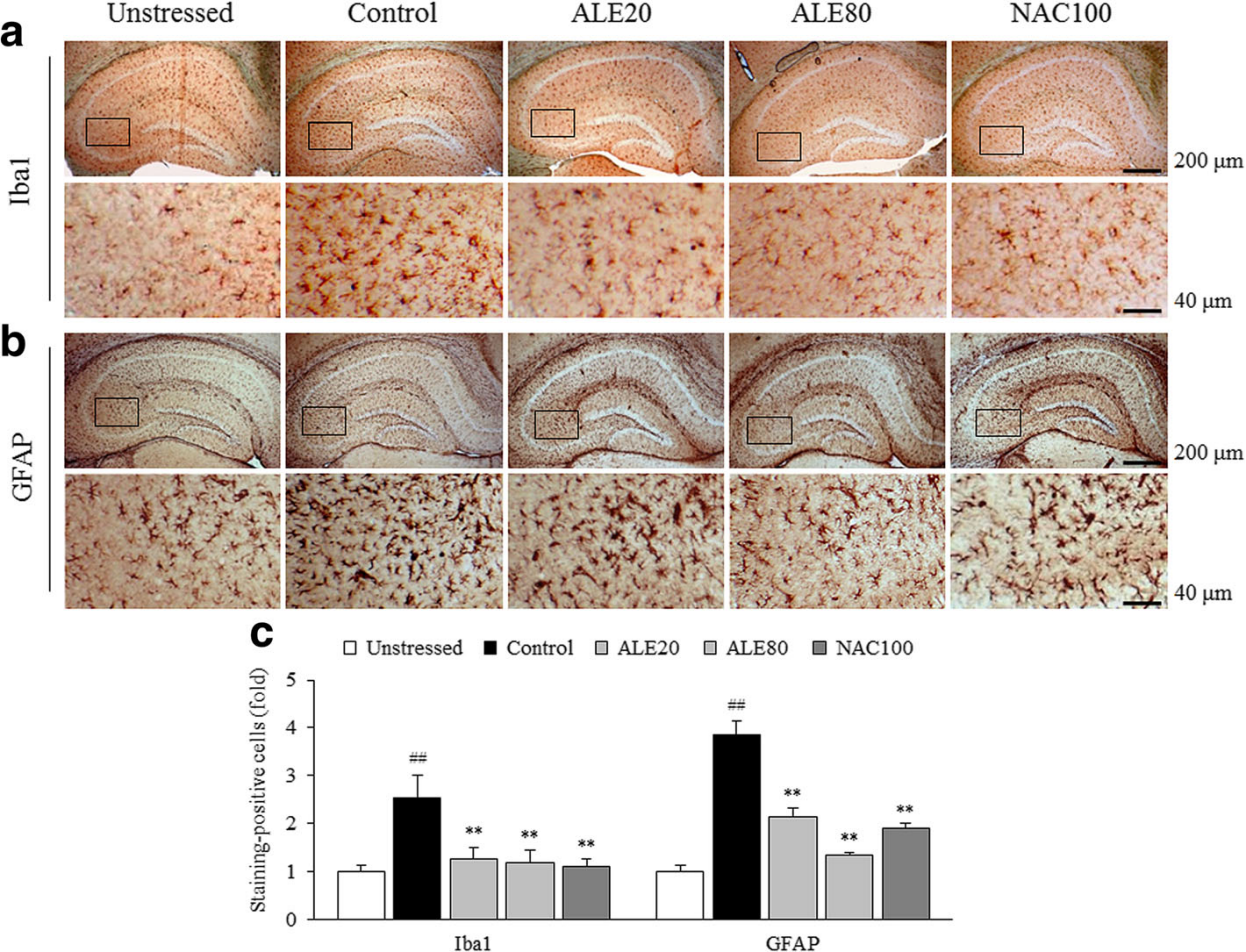
Immunohistochemical findings. Iba1-positive microglial cells and GFAP-positive astrocytes were stained in the hippocampus. Representative photomicrographs were taken at magnifications of 40× and the CA3 region was magnified at 200× (**a**, **b**). Then, the intensity of Iba1- and GFAP-positive staining was quantified (**c**). The data are expressed as the means ± SD (*n* = 3). ^##^ *P* < 0.01 compared with the unstressed group; ^**^ *P* < 0.01 compared with the control group. CA3; cornu ammonis 3

Repeated restraint stress activated the hippocampal microglial cells and astrocytes, as evidenced by striking elevations of Iba1 and GFAP signals in the cornu ammonis 3 (CA3) region of hippocampus compared with the unstressed group (Fig. 3a and b). These activations were considerably and significantly attenuated by ALE treatment when compared with the control group in quantitative analyses on positive cells (*P* < 0.01 for both, Fig. 3c). NAC treatment also showed similar effects on these activations.

### Effects on TNF-α and IL-1β in the hippocampus

**Fig. 4:**
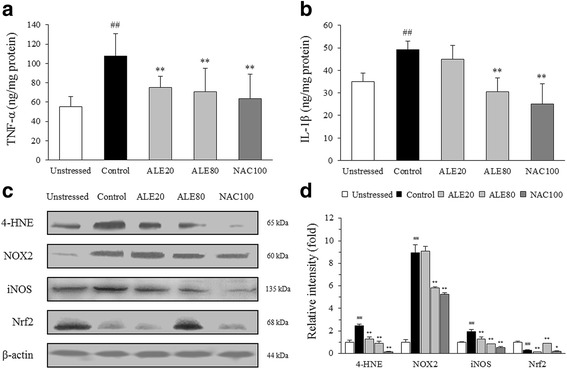
Pro-inflammatory cytokine levels and western blot analyses in the hippocampus. TNF-α and IL-1β levels in the hippocampus were determined using ELISA (**a**, **b**). 4-HNE-modified protein, NOX2, iNOS and Nrf2 concentrations in the hippocampus were determined by western blotting (**c**). Then, the intensities of 4-HNE, NOX2, iNOS and Nrf2 protein expression were quantified (**d**). The data are expressed as the means ± SD (*n* = 7). ^##^ *P* < 0.01 compared with the unstressed group; ^*^ *P* < 0.05 and ^**^ *P* < 0.01 compared with the control group

Repeated restraint stress elevated the hippocampal concentration of TNF-α and IL-1β by 1.9-fold and 1.4-fold compared with the unstressed group, which was significantly ameliorated by ALE treatment (*P* < 0.01 for TNF-α and *P* < 0.01 for IL-1β in only 80 mg/kg group, Fig. 4a and b). NAC treatment also significantly reduced the elevations of these cytokines.

### Western blot analysis in the hippocampus

Repeated restraint stress dramatically activated the hippocampal 4-HNE-modified protein, NOX2 and iNOS but reduced the Nrf2 protein quantities in the hippocampus, which was significantly alleviated by ALE (80 mg/kg) treatment compared with the control group (*P* < 0.01 for 4-HNE, NOX2, iNOS and Nrf2, Fig. 4c and d). NAC treatment notably attenuated the expression of 4-HNE and iNOS in the hippocampus.

### Effects on antioxidants in the hippocampus

**Fig. 5:**
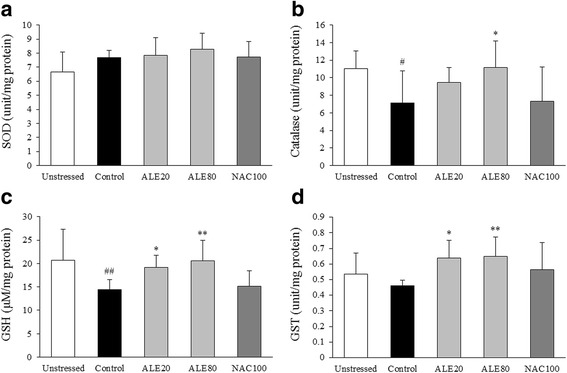
Antioxidants in the hippocampus. SOD (**a**), catalase (**b**), GSH (**c**), and GST (**d**) levels in the hippocampus were determined using ELISA. The data are expressed as the means ± SD (*n* = 7). ^#^ *P* < 0.05, ^##^ *P* < 0.01 compared with the unstressed group; ^*^ *P* < 0.05, ^**^ *P* < 0.01 compared with the control group

Repeated restraint stress exhausted hippocampal antioxidants, especially GSH (0.7-fold) and catalase (0.6-fold) in the hippocampus when compared with the unstressed group. These alterations were significantly restored by ALE treatment (*P* < 0.05 or <0.01 for GSH and *P* < 0.05 for catalase; Fig. 5b and c). The hippocampal SOD and GST levels were not significantly reduced following repeated restraint stress. However, the GST levels were significantly increased by ALE treatment (*P* < 0.05 or <0.01, Fig. 5a and d). NAC treatment did not show any significant changes in the above biomarkers.

### Effects on NF-κB activation in BV2 microglial cell

**Fig. 6:**
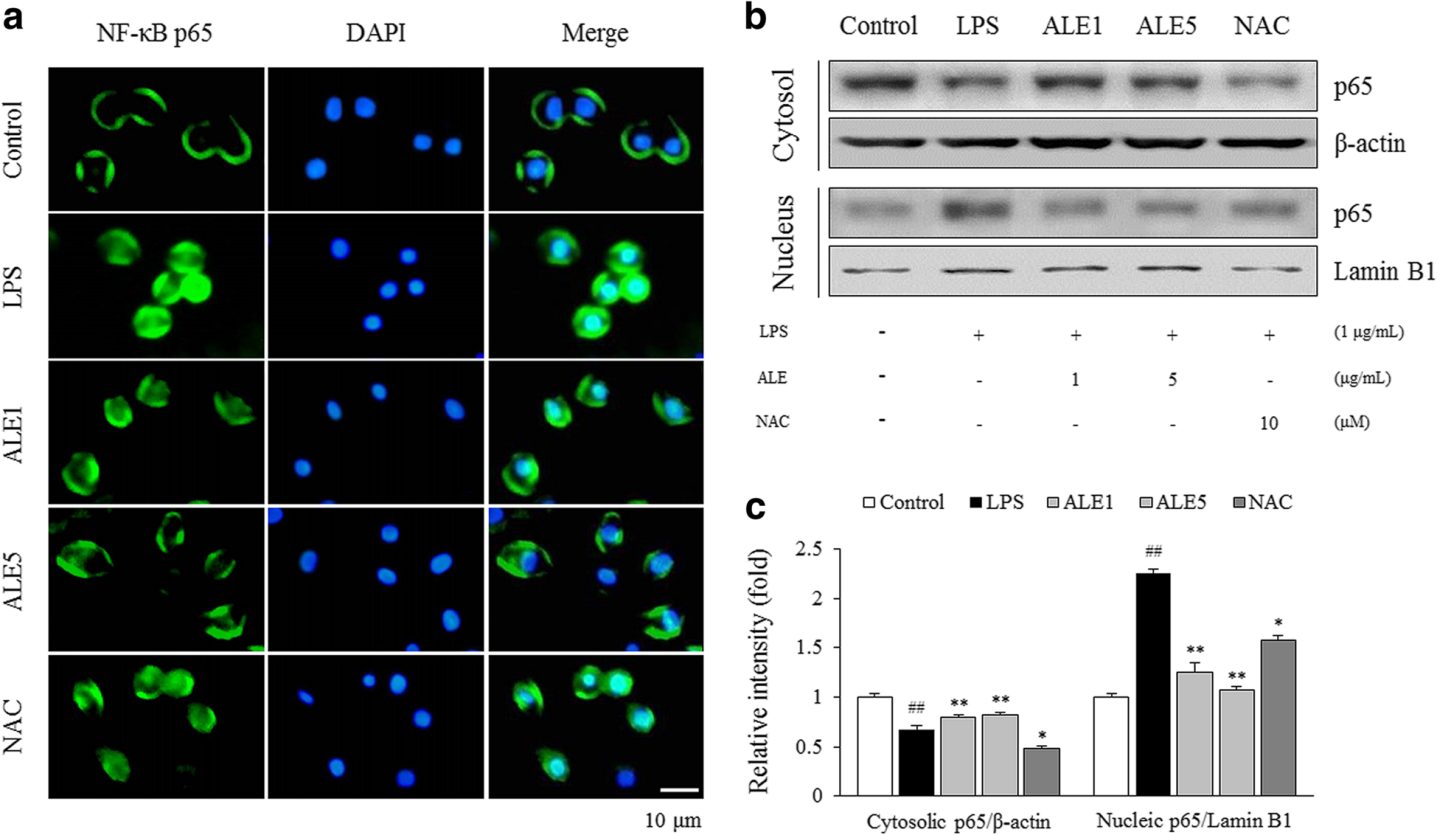
NF-kappa B (NF-κB) activation in lipopolysaccharide (LPS)-treated BV2 microglial cells. Cells were pre-treated with vehicle, ALE (1 and 5 μg/mL) or NAC (10 μM) for 3 h before 1 h exposure to LPS (1 μg/mL). Immunofluroscent staining shows the cellular distribution of NF-κB p65 subunit (green) in microglia. The nuclei were counterstained with DAPI (blue) (**a**). The p65 expressions in cytosolic and nucleic extracts, respectively, were determined by western blot and semi-quantified using Image J (**b**, **c**). The data are obtained from three independent experiments and expressed as the means ± SD (*n* = 3). ^##^ *P* < 0.01 compared with control group; ^*^ *P* < 0.05, ^**^ *P* < 0.01 compared with LPS group

LPS (1 μg/mL) treatment activated microglial NF-κB, resulting in translocation of p65 subunit to the nucleus. ALE (5 μg/mL) pre-treatment, however, dramatically inhibited NF-κB p65 nuclear translocation (Fig. 6a). Western blot analysis of the cytoplasmic and nuclear extracts also supported the immunofluorescence data. LPS stimulation resulted in 0.6-fold decrease and 2.3-fold increase in cytosolic and nucleic p65 levels, respectively, which was significantly normalized by ALE treatment (*P* < 0.01 for both, Fig. 6b and c). NAC, however, did not regulate NF-κB activation by LPS.

## Discussion

Under uncontrolled chronic stress, the negative feedback system of the HPA axis becomes disrupted, resulting in a prolonged activation of the HPA axis [32]. The brain is the main target of excessive stress, which develops stress-related psychiatric disorders and cognitive dysfunction [33, 34]. In particular, hippocampus, amygdala, and prefrontal cortex tissues are susceptible to long-term stress and undergo functional or structural remodeling in response [35, 36]. One recent study showed that restraint stress induced morphological changes in the blood brain barrier, supporting the linkage of stress to neurodegenerative disorders [37]. To investigate the pharmaceutical potential of *A. Lignum* for anti-oxidative protection against brain injury by stress, the present study adopted a mouse restraint stress model.

The restraint model is the most commonly employed methodology for the induction of stress in experimental animals [26]. As expected, repeated restraint stresses for 6 h every 11 day evoked significant elevations of two typical stress hormones, corticosterone and adrenaline, in the serum (Fig. 2a). Glucocorticoids, as lipophilic steroids, can enter the blood-brain barrier and bind to either of the two types of receptors: high-affinity mineralocorticoid receptors or low-affinity glucocorticoid receptors [38]. Contrary to mineralocorticoid receptors, the glucocorticoid receptors are activated to a larger extent when glucocorticoid levels rise under chronic stress [39, 40]. High concentrations of glucocorticoid are known to trigger mitochondrial dysfunction, cellular apoptosis, synaptic alterations, neuroinflammatory processes, and epigenetic changes in brain [41–44].

Among various brain regions, the hippocampus plays key roles in short- and long-term memory and is highly susceptible to oxidative stress [45, 46]. Many clinical studies observed degenerative changes in the hippocampal region of patients with learning and memory deficits such as Alzheimer’s disease [47, 48]. The hippocampus contains glucocorticoid receptors in high proportions [49]. Moreover, corticosterone is known to accelerate ROS formation in brain tissue via mitochondrial activation, which is further aggravated by adrenaline and noradrenaline actions [50, 51]. Additionally, chronic stress promotes NO overproduction in the brain and can suppress hippocampal neurogenesis [52, 53]. In our results, ALE treatment showed anti-oxidative brain injury effects by significantly attenuating the elevation of serum corticosterone and adrenaline levels and oxidative stress parameters, including ROS, NO, and MDA (Fig. 2b–d). These conclusions were partially supported by data measuring antioxidant components, including GSH, GST, and catalase (Fig. 5b–d) and western blot analyses for 4-HNE, iNOS and Nrf2 in the hippocampus (Fig. 4c). These results indicate that ALE protects the hippocampus from stress-induced oxidative alteration.

Brain tissue is prone to oxidative stress due to its compositional and functional features, such as excessive consumption of oxygen [54]. Furthermore, oxidative stress is a potent risk factor in brain ageing and neurodegenerative disorders [55, 56]. Interestingly, many animal studies found that phagocytic NOX2 isoform mediates brain oxidative injury by chronic stress and contributes to behavioral alterations, including anxiety- and depression-like behaviors [8, 57, 58]. The hippocampal region especially showed vulnerability to NOX-derived ROS [9]. In our study, repeated restraint stress notably activated hippocampal NOX2 expression, which was significantly ameliorated by ALE (80 mg/kg) treatment (Fig. 4c and d). The involvement of NOX2 in hippocampal oxidative stress is also partially supported by our histopathological findings showing the significant activation of microglial cells, i.e., the resident immune cells of the central nervous system in the hippocampus (Fig. 3a).

Neuroinflammation is one of the main mechanisms in stress-related brain disorders and is closely linked to oxidative stress [15, 59]. Under chronic stress, microglia cells become substantially activated and release nitric oxide and pro-inflammatory cytokines, including TNF-α and IL-1β [60, 61]. One study demonstrated that the hippocampal CA3 region is one of the most stress-responsive areas with significant microglial alterations [62]. NOX2-derived ROS also plays a role in astrogliosis, which indicates NOX2 and oxidative by-products to be critical factors for glial cell activation and pro-inflammatory cytokine release [63]. As expected, repeated restraint stress drastically activated both microglia and astrocytes, showing ramified cellular processes in the hippocampus, especially in the CA3 region (Fig. 3). However, administration with ALE significantly suppressed neuroglial cell activation and led to a dramatic decrease in pro-inflammatory cytokines, including TNF-α and IL-1β (Fig. 4a and b). Meanwhile, NF-κB is a main transcriptional factor for production of pro-inflammatory cytokines [64]. One main hypothesis formulates psychological stress induces inflammatory responses via Toll-like receptor 4 activation, similar to infection process [65]. In this study, we treated BV2 microglial cell line with LPS to simulate neuroinflammatory state. Interestingly, ALE pre-treatment significantly blocked NF-κB p65 translocation to the nucleus (Fig. 6), which accounts for ALE’s anti-inflammatory effects in the brain exposed to repeated stress. These results suggest that ALE could regulate the production of free radicals and pro-inflammatory cytokines via modulating microglial and astrocytic activation, and NF-κB signaling pathway.

*A. Lignum* is fragrant resinous heartwood of the *Aquilaria* species (family Thymelaeaceae) mainly found in Southeast Asian tropical regions [66]. For a long time, Asians have considered the plant as a priceless natural product with anxiolytic, sedative, and anti-inflammatory effects [67]. Recently, various *Aquilaria* species have been scientifically shown to possess anti-oxidative, anti-inflammatory, and anxiolytic properties [25, 68, 69]. Our preliminary in vitro study also revealed that ALE significantly decreased ROS and LDH activities, which corresponds with its anti-oxidative effect in the current data. The active compounds responsible for the pharmaceutical actions of *A. Lignum* have not been fully elucidated yet. According to one study, trans-feruloyltyramine, one compound of *A. Lignum,* significantly attenuates β-amyloid peptide-induced neurotoxicity by reducing ROS and deactivating apoptotic cell death [70]. Studies for identifying its main active compounds will be conducted in the future.

To our knowledge, this study is one of the first to report the efficacy of *A. Lignum* on preventing repeated restraint stress-induced hippocampal oxidative stress. The activity of 30% ethanol extract of *A. Lignum* (80 mg/kg) was generally similar or superior to 100 mg/kg of NAC, an amino acid precursor to GSH. Interestingly, NAC treatment notably inactivated iNOS but failed to up-regulate hippocampal Nrf2, a key transcription factor for antioxidants (Fig. 4c). We speculate such factors led to NAC’s inconsistent anti-oxidative effect.

In further studies, we need to verify active single compound of *A. Lignum* using liquid chromatographic fractionation and its action mechanisms, especially regarding the passage through the blood-brain barrier.

## Conclusion

*A. Lignum* exhibits protective properties against hippocampal oxidative and inflammatory alterations under chronic stress. Its putative corresponding mechanisms involve the modulation in ROS and pro-inflammatory cytokines by microglial cells, and the release of stress hormones.

## Abbreviations

ANOVA: Analysis of variance
GSH: Glutathione
GST: Glutathione S-transferase
HNE: Hydroxynonenal
IL: Interleukin
INOS: Inducible nitric oxide synthase
LPS: Lipopolysaccharides
MDA: Malondialdehyde
MS: Mass spectrometry
NF-κB: Nuclear factor kappa B
NO: Nitric oxide
NOX: NADPH oxidase
Nrf: Nuclear factor (erythroid-derived 2)-like
RIPA: Radioimmunoprecipitation assay
ROS: Reactive oxygen species
SOD: Superoxide dismutase
TNF: Tumor necrosis factor
UHPLC: Ultra-high performance liquid chromatography
